# From Susceptibility to Disease Severity: Re-Discovering the Clinical Role of *MMP-12* Genotypes in Asthma

**DOI:** 10.3390/life16071211

**Published:** 2026-07-22

**Authors:** Shou-Cheng Wang, Te-Chun Hsia, Te-Chun Shen, Liang-Wen Hang, Jie-Long He, Ding-Han Chen, Kai-Ling Huang, Chia-Wen Tsai, Wen-Shin Chang, Da-Tian Bau

**Affiliations:** 1 Taichung Armed Forces General Hospital, Taichung 411228, Taiwan; 2 Department of Medical Planning, Medical Affairs Bureau, Ministry of National Defense, Taipei 104237, Taiwan; 3 National Defense Medical University, Taipei 114201, Taiwan; 4 Division of Pulmonary and Critical Care Medicine, Department of Internal Medicine, China Medical University Hospital, Taichung 404327, Taiwan; 5 Terry Fox Cancer Research Laboratory, Department of Medical Research, China Medical University Hospital, Taichung 404327, Taiwan; 6 School of Medicine, College of Medicine, China Medical University, Taichung 404333, Taiwan; 7 Division of Critical Care Medicine, Chu Shang Show Chwan Hospital, Nantou 55782, Taiwan; 8 Department of Post-Baccalaureate Veterinary Medicine, Asia University, Taichung 413305, Taiwan; 9 Graduate Institute of Biomedical Sciences, China Medical University, Taichung 404333, Taiwan; 10 Office of Research and Development, Asia University, Taichung 413305, Taiwan

**Keywords:** age, asthma, gender, genotype, polymorphism, matrix metalloproteinase-12, severity

## Abstract

Asthma is a chronic inflammatory airway disorder influenced by genetic and environmental factors. Matrix metalloproteinase-12 (MMP-12) plays a key role in airway remodeling and inflammation, but its genetic contribution to asthma remains unclear. This study investigated the associations of *MMP-12* rs2276109 and rs652438 polymorphisms with asthma susceptibility and severity in 198 Taiwanese asthma patients and 453 controls. Genotypes were determined by PCR-RFLP, and odds ratios (ORs) with 95% confidence intervals (CIs) were calculated using logistic regression. Neither *MMP-12* rs2276109 nor rs652438 was significantly associated with overall asthma susceptibility. For rs2276109, the CT and CC genotypes yielded ORs of 1.06 (95% CI = 0.56–2.00, *p* = 0.9913) and 6.98 (95% CI = 0.72–67.59, *p* = 0.0857), respectively. However, among individuals aged 25–40 years, rs652438 CT and CC genotypes were associated with elevated asthma risk (OR = 1.86, 95% CI = 1.14–3.04, *p* = 0.0100; OR = 3.60, 95% CI = 1.11–11.65, *p* = 0.0494). Furthermore, *MMP-12* rs652438 was significantly associated with asthma severity under both the CT genotype (OR = 2.32, 95% CI = 1.16–4.62, *p* = 0.0250) and dominant models (OR = 2.45, 95% CI = 1.28–4.72, *p* = 0.0106). Although *MMP-12* polymorphisms do not appear to influence overall asthma susceptibility, rs652438 may serve as a genetic marker for disease severity and clinical stratification in asthma.

## 1. Introduction

Asthma is a chronic respiratory disorder characterized by persistent airway inflammation, reversible airflow limitation, bronchial hyperresponsiveness, and progressive remodeling of airway structures [1,2]. Current estimates indicate that nearly 300 million individuals are affected worldwide, and epidemiological projections suggest that the number of cases may rise by an additional 100 million in the near future [3,4]. From a molecular epidemiological viewpoint, genetic predisposition is recognized as a critical determinant of asthma susceptibility and disease heterogeneity. Numerous investigations have demonstrated that polymorphic alterations in immune- and inflammation-associated genes significantly modulate both asthma risk and therapeutic responsiveness. Mounting evidences have shown that cytokine signaling pathway genotypes have been involved in the regulation of airway inflammation, remodeling, and immune dysregulation, for instance, the genotypes of *vascular endothelial growth factor* (*VEGF*) [5], *interleukin-4* (*IL-4)* [6,7,8], *IL-12* [9], *IL-13* [10,11,12], and the metalloprotease-coding gene, a disintegrin and metalloproteinase-33 (*ADAM-33*) [13,14]. Nevertheless, asthma development cannot be attributed solely to inherited genetic factors, as environmental exposures also exert profound influences on disease initiation and progression. Increasing evidence indicates that interactions between susceptible genotypes and external stimuli are central to asthma pathogenesis. Environmental risk factors, including aeroallergen exposure [15,16], airborne fine particulate matter (PM2.5) [17,18], and cigarette smoke [19,20], are capable of amplifying inflammatory cascades and exacerbating airway hypersensitivity.

Among the numerous molecular mediators involved in asthma pathogenesis, matrix metalloproteinases (MMPs) have attracted considerable attention because of their capacity to degrade extracellular matrix (ECM) components and regulate inflammatory signaling pathways [21,22]. The MMP family comprises more than 25 proteolytic enzymes, each characterized by distinct substrate specificities and biological functions [23]. Although substantial research has focused on MMP-2 and MMP-9 in respiratory diseases, the contribution of MMP-12 has received comparatively less investigation. MMP-12, also referred to as macrophage elastase or matrixin, has recently emerged as an important mediator linking multiple pathological processes associated with pulmonary disorders. Increasing evidence suggests that MMP-12 participates in airway inflammation [24], ECM remodeling [25], and airway hyperresponsiveness [26], all of which are central features in the development and progression of asthma. Notably, Warner and his colleagues first proposed in 2004 that MMP-12 contributes not only to acute allergen-induced inflammatory responses but also to chronic structural remodeling within pulmonary tissues [27]. Subsequent investigations have further demonstrated that multiple inflammatory and structural cell populations are capable of producing MMPs, including MMP-12, during asthmatic responses. Among these, macrophages are considered one of the principal cellular sources and play critical roles in orchestrating airway inflammation and tissue remodeling [28,29].

The *MMP-12* gene, located on chromosome 11q22.2 and composed of 10 exons separated by nine introns, harbors several single nucleotide polymorphisms (SNPs) that may alter transcriptional regulation or enzymatic properties of its encoded protein [30]. Among these variants, rs2276109 has attracted attention because the A allele enhances the binding affinity of activator protein-1 (AP-1) to the promoter region, thereby increasing *MMP-12* transcriptional activity [31,32]. In contrast, the rs652438 polymorphism results in a nonsynonymous amino acid substitution from asparagine to serine within the hemopexin-like domain, an important structural region involved in substrate recognition and proteolytic function [33,34]. At the biochemical level, MMP-12 is initially synthesized as a latent 54 kDa zymogen that subsequently undergoes autocatalytic processing to produce active isoforms of approximately 45 and 22 kDa. Once activated, MMP-12 exhibits broad-spectrum proteolytic activity against numerous extracellular matrix constituents, including fibronectin, elastin, vitronectin, laminin, type IV collagen, and heparan sulfate [35,36,37]. Through degradation of these structural components, MMP-12 may contribute to airway remodeling and structural alterations. Importantly, the biological functions of MMP-12 extend beyond its own matrix-degrading capacity. Previous studies have demonstrated that MMP-12 can further activate additional MMPs, including MMP-2 and MMP-3, thereby potentiating downstream proteolytic cascades and amplifying pathological remodeling processes [38].

**Figure 1:**
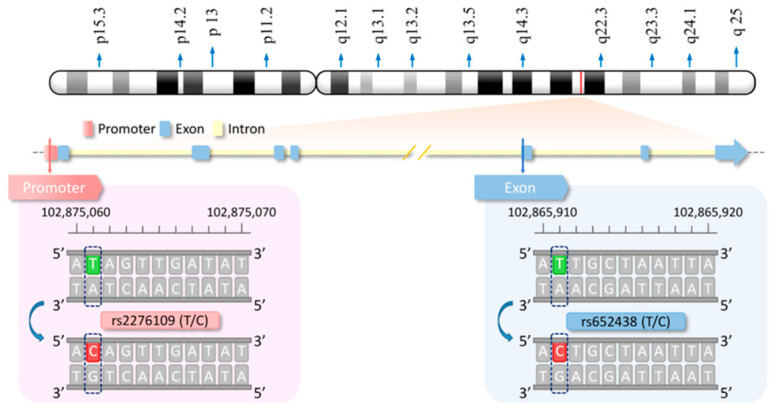
Physical map for *MMP-12* rs2276109 and rs652438 polymorphic sites. The consequences of polymorphic alterations from T to C of both locus are shown.

Briefly, all the evidence above strongly implies that genetic variations in *MMP-12* may play a role in modulating inflammatory responses and ECM turnover during the pathogenesis of pulmonary disorders, particularly asthma. To our surprise, despite the growing recognition of MMP-12 in airway inflammation and remodeling, only three published studies have evaluated the relationship between *MMP-12* polymorphisms and asthma susceptibility in global human populations, including cohorts from the United Kingdom, Bulgaria, and Japan [29,39,40]. That is to say, evidence regarding the contribution of *MMP-12* genetic variants to asthma risk remains largely unknown, particularly in Asian populations. To address this knowledge gap, the present study aimed to explore the potential involvement of *MMP-12* polymorphisms, rs2276109 and rs652438 (illustrated in Figure 1), in modulating susceptibility to asthma within a representative Taiwanese cohort containing 198 asthma patients and 453 non-asthmatic subjects. In addition, we also aimed to evaluate the interactions between these *MMP-12* genetic variants and important demographic as well as clinical parameters, including age, sex, and disease severity, in order to determine whether *MMP-12* genotypes may serve as potential predictive biomarkers for asthma susceptibility and prognostic outcomes.

## 2. Materials and Methods

### 2.1. Recruitment of Asthmatic Patients and Control Participants

This study was conducted in accordance with the ethical principles of the Declaration of Helsinki and was approved by the Institutional Review Board of China Medical University Hospital (CMUH106-REC1-004). Written informed consent was obtained from all participants or their legal guardians before enrollment. A total of 198 adult patients with asthma and 453 non-asthmatic controls were consecutively recruited at China Medical University Hospital between 2008 and 2010, following the recruitment strategies established in our previous study [41,42]. All participants were ethnic Taiwanese and were older than 25 years. Participants were stratified into two age groups (25–40 years and >40 years) to distinguish younger and older adult patients according to the predefined study design and because no participants were younger than 25 years. Asthma treatment categories were assigned according to the contemporaneous GINA recommendations available during patient recruitment (2008–2010). These categories reflected treatment intensity required to achieve asthma control rather than the current GINA severity classification [43]. The inclusion criteria for asthma patients were: (1) more than two or three episodes of wheezing and shortness of breath during the previous year; (2) physician-confirmed asthma with reversible and variable airflow obstruction demonstrated by spirometry; (3) compatible clinical symptoms; and (4) prescription of asthma medications. There was no sex restriction for patient recruitment. Asthma severity was classified into four groups according to the level of treatment required to achieve symptom control and prevent exacerbations, following the GINA recommendations. Patients receiving as-needed inhaled corticosteroid (ICS)-formoterol alone were classified as Group 1 (mildest); those receiving low-intensity maintenance controller therapy (ICS-formoterol, leukotriene receptor antagonists, or chromones) were classified as Group 2; those receiving low-dose ICS-long-acting β2 agonist (LABA) therapy were classified as Group 3; and those receiving high-dose ICS-LABA therapy were classified as Group 4 (most severe). Because the number of patients in each treatment category was limited, Groups 1–2 and Groups 3–4 were combined for exploratory statistical analyses to improve statistical stability. The control subjects were frequency-matched to asthma patients by age (±5 years) and sex after random selection from the Health Examination Cohort of China Medical University Hospital. The inclusion criteria for controls were: (1) no physician-diagnosed asthma or other chronic pulmonary diseases, including COPD; (2) no history of wheezing, shortness of breath, or other symptoms suggestive of allergic diseases; (3) no use of asthma medications; (4) no first-degree relatives with a history of asthma; and (5) age older than 25 years. Therefore, the non-asthmatic status of the control subjects was confirmed based on their medical history, physician assessment, and the absence of asthma-related symptoms and medication use. Smoking status was recorded at enrollment and included in the baseline characteristics as well as the multivariable logistic regression analyses. Detailed demographic and clinical characteristics of all participants are summarized in Table 1.

### 2.2. DNA Extraction and Sample Preservation

Genomic DNA was extracted from the buffy coat fraction of peripheral blood specimens obtained from all enrolled participants, and sample processing was completed within 24 h after blood collection to ensure optimal nucleic acid quality and stability [44,45,46]. The purified genomic DNA samples were subsequently preserved at −80 °C until further analysis. To ensure analytical consistency and minimize inter-sample variation, DNA specimens from asthmatic patients and control individuals were standardized to equivalent concentrations, aliquoted into working stocks, and systematically archived according to protocols established in our previous asthma-related investigations [47,48]. The prepared DNA aliquots were arranged in 96-well plates and maintained at −20 °C prior to genotyping analyses.

### 2.3. MMP-12 Genotyping Methodology

Two *MMP-12* polymorphisms, rs2276109 and rs652438, were analyzed using distinct genotyping approaches. Both the rs2276109 and rs652438 variants were determined by polymerase chain reaction–restriction fragment length polymorphism (PCR-RFLP) analysis. For amplification of the rs2276109 region, the primer sequences were 5′-GAGATAGTCAAGGGATGATATCA-3′ (forward) and 5′-AAGAGCTCCAGAAGCAGTGG-3′ (reverse). As for rs652438, it was amplified using the primer pair 5′-GGATAATTTGGCTCTGGTCTTAC-3′ (forward) and 5′-CCATGGGAACCATAGAAAAGA-3′ (reverse). Specifically, a deliberate nucleotide mismatch C was introduced into the forward primer sequence of rs652438 to create a restriction enzyme recognition site, thereby enabling discrimination of the alleles by PCR-RFLP analysis. Following PCR amplification, rs2276109 and rs652438 amplicons were digested with *Pvu* II and *Mfe* I-HF (high fidelity, New England BioLabs, Ipswich, MA, USA), and the digestion adducts were subsequently resolved on 3% agarose gels. Genotypes were assigned according to the characteristic restriction fragment patterns; the rs2276109 TT genotype generated a single 199 bp fragment, the CT genotype produced three fragments of 199, 175, and 24 bps, whereas the CC genotype yielded two fragments of 175 and 24 bps. The rs652438 CC genotype generated a single 204 bp fragment, the CT genotype produced three fragments of 204, 181, and 23 bps, whereas the TT genotype yielded two fragments of 181 and 23 bps. Genotypes were assigned according to the presence or absence of the major restriction fragments. Because the 23 bp and 24 bp digestion products could not be reliably visualized on a standard 3% agarose gel, genotype determination was based primarily on the conversion of the 199 bp fragment to the 175 bp fragment for rs2276109 and the 204 bp fragment to the 181 bp fragment for rs652438. To ensure the reliability and reproducibility of the genotyping analyses, all experiments were independently performed by at least two well-trained investigators under blinded conditions. Notably, complete agreement was achieved between the independent assessments, with a concordance rate of 100%, thereby confirming the accuracy and consistency of the genotyping procedures.

### 2.4. Statistical Analysis Methodology

The genotypic distributions of the *MMP-12* polymorphisms in the control group were first assessed for conformity with Hardy–Weinberg equilibrium (HWE) using the chi-square goodness-of-fit test to confirm the representativeness and genetic stability of the investigated population. Differences in age between asthmatic patients and non-asthmatic controls were analyzed using the unpaired *Student’s t*-test. In addition, variations in demographic characteristics, including age and sex, as well as genotypic and allelic frequencies between asthma cases and controls, were evaluated using Pearson’s chi-square test based on appropriate 2 × 2 or 2 × 3 contingency table analyses. To determine the relationship between individual *MMP-12* polymorphisms and asthma susceptibility, odds ratios (ORs) and their corresponding 95% confidence intervals (CIs) were estimated using logistic regression analyses. Furthermore, the Cochran–Armitage trend test was applied to assess genotype–asthma associations under an additive genetic inheritance model. The Haldane–Anscombe correction was used for OR and 95% CI calculation when the number of specific cells is zero. Both dominant and recessive inheritance models were additionally evaluated, and the results are summarized in Table 2. All statistical analyses were conducted using two-tailed tests, and a *p*-value below 0.05 was considered indicative of statistical significance.

## 3. Results

### 3.1. Genotypic Distribution of MMP-12 Polymorphisms Among the Cohort

Table 2 presents the genotype distributions of *MMP-12* rs2276109 and rs652438 polymorphisms among the 198 asthmatic patients and 453 non-asthmatic controls. Analysis using the chi-square goodness-of-fit test confirmed that both polymorphisms conformed to HWE in the control group (*p* = 0.6819 and 0.2609 for rs2276109 and rs652438, respectively). No statistically significant differences were observed in rs2276109 genotype frequencies between asthmatic cases and non-asthmatic controls (*p* for trend = 0.2622). Compared to the wild-type TT genotype, the CT and CC genotypes were associated with ORs of 1.06 (95% CI = 0.56–2.00, *p* = 0.9913) and 6.98 (95% CI = 0.72–67.59, *p* = 0.0857), respectively. In the recessive genetic model, the associations did not reach statistical significance for the CC genotype compared with the TT + CC genotype (OR = 6.95, 95% CI = 0.72–67.27, *p* = 0.0862). On the contrary, under the dominant model (CT + CC vs. TT), an absolutely non-significant association with asthma risk was found (OR = 1.23, 95% CI = 0.68–2.24, *p* = 0.5966). For the rs652438 polymorphism, consistent findings across co-dominant, recessive and dominant genetic models indicated no statistically significant association with asthma risk (Table 2).

### 3.2. Allelic Frequency Distributions of MMP-12 Polymorphisms Among the Cohort

To complement the genotypic analyses, allelic frequency distributions for *MMP-12* rs2276109 and rs652438 were examined, and the results are shown in Table 3. The variant C allele of rs2276109 appeared slightly more frequently in the asthmatic group (5.3%) than in the non-asthmatic group (3.9%), yielding an OR of 1.39 (95% CI = 0.80–2.43, *p* = 0.3032), indicating no significant association. Similarly, allelic frequencies for rs652438 did not differ significantly between asthmatic patients and non-asthmatic controls, with an OR of 1.40 (95% CI = 0.99–1.97, *p* = 0.0711). These findings collectively suggest that neither *MMP-12* rs2276109 nor rs652438 genotypes exhibited a significant impact on susceptibility to asthma within the investigated Taiwanese cohort.

### 3.3. Association of MMP-12 rs652438 Genotypes with Age in Asthma Risk Prediction

To further investigate whether age modified the association between *MMP-12* rs652438 polymorphisms and asthma susceptibility, stratified analyses were conducted according to participant age (25–40 years versus >40 years), and the results are summarized in Table 4. Age-stratified analyses were performed as exploratory analyses to evaluate potential effect modification. Among younger individuals aged 25–40 years, the CT genotype was significantly associated with an elevated risk of asthma compared with the wild-type TT genotype, with an OR of 1.86 (95% CI = 1.14–3.04, *p* = 0.0100). Moreover, carriers of the CC genotype exhibited an even higher asthma susceptibility, with an OR of 3.60 (95% CI = 1.11–11.65, *p* = 0.0494). Consistently, the Cochran–Armitage trend test demonstrated a significant genotype-dependent trend in asthma risk among younger subjects (*p* for trend = 0.0014). In contrast, no significant association was observed between rs652438 genotypes and asthma susceptibility in individuals older than 40 years (all *p* > 0.05), despite a borderline significant trend analysis (*p* for trend = 0.0487). To sum up, the findings suggest that the influence of *MMP-12* rs652438 polymorphisms on asthma susceptibility may be more obvious in younger populations (Table 4).

### 3.4. Association of MMP-12 rs652438 Genotypes with Gender in Asthma Risk Prediction

We next evaluated the potential interaction between *MMP-12* rs652438 polymorphisms and gender in relation to asthma susceptibility, and the results are shown in Table 5. Gender-stratified analyses were performed as exploratory analyses to evaluate potential effect modification. No statistically significant associations were identified between rs652438 genotypes and asthma risk among either male or female participants. Specifically, in males, the CT and CC genotypes were associated with ORs of 1.42 (95% CI = 0.75–2.67, *p* = 0.3647) and 2.53 (95% CI = 0.61–10.46, *p* = 0.2346), respectively, compared with the TT genotype. Similarly, among females, the CT and CC genotypes yielded ORs of 1.23 (95% CI = 0.72–2.11, *p* = 0.5295) and 1.81 (95% CI = 0.40–8.28, *p* = 0.4257), respectively. Furthermore, trend analyses revealed no significant genotype-dependent associations in either males or females (*p* for trend = 0.1112 and 0.3036, respectively). These findings indicated that gender does not appear to significantly modify the association between *MMP-12* rs652438 polymorphisms and asthma susceptibility in this Taiwanese cohort (Table 5).

### 3.5. Association of MMP-12 rs652438 Genotypes with Asthma Severity

To further clarify the clinical significance of *MMP-12* rs652438 polymorphisms, their associations with asthma severity were examined among asthmatic patients, and the results are summarized in Table 6. Asthma severity-stratified analyses were performed as exploratory analyses to evaluate potential effect modification. Compared with individuals carrying the wild-type TT genotype, asthmatic patients harboring the CT genotype exhibited a significantly increased likelihood of developing more severe asthma, with an OR of 2.32 (95% CI = 1.16–4.62, *p* = 0.0250). Although carriers of the CC genotype demonstrated an elevated OR of 3.53 (95% CI = 0.75–16.49), this association did not reach statistical significance, likely due to the limited sample size. Importantly, trend analysis revealed a significant dose-dependent association between rs652438 variant genotypes and asthma severity (*p* for trend = 0.0059). Under the dominant genetic model, individuals carrying the combined CT + CC genotypes had a 2.45-fold higher odds of more severe asthma compared with TT carriers (95% CI = 1.28–4.72, *p* = 0.0106). In contrast, no significant association was observed under the recessive model (CC versus TT + CT, *p* = 0.2201). Overall, these findings suggest that the rs652438 variant C allele, particularly in CT + CC carriers, may contribute to increased asthma severity and could serve as a potential genetic marker associated with asthma severity that warrants further validation in larger, multicenter, prospective studies. (Table 6).

## 4. Discussion

MMP-12, also known as macrophage elastase, is a zinc-dependent proteolytic enzyme primarily secreted by macrophages, airway epithelial cells, and airway smooth muscle cells. Increasing evidence indicates that MMP-12 plays a pivotal role in asthma pathogenesis through modulation of airway inflammation, ECM remodeling, mucus hypersecretion, and airway hyperresponsiveness. Chiba and his colleagues first demonstrated that MMP-12 expression was markedly increased in a rat model of allergic bronchial asthma [28]. Importantly, MMP-12 mRNA expression increased during the early phase after allergen challenge, whereas enzymatic activity peaked later during established airway inflammation, suggesting that MMP-12 participates in both initiation and progression of allergic airway responses [28]. Subsequent pathological studies further linked MMP-12 to airway remodeling in human asthma. Araujo and his colleagues investigated ECM components within airway smooth muscle in fatal asthma and found a significant MMP-12 overexpression in large airways of fatal asthma patients compared with controls [49]. Elevated MMP-12 was associated with increased fibronectin deposition and altered ECM composition, supporting the concept that MMP-12 contributes to the degradative microenvironment responsible for airway structural remodeling in severe asthma [49]. Di Valentin and his colleagues found that MMP-12 remained highly overexpressed throughout asthma progression in an ovalbumin-induced asthma animal model [50]. The authors proposed MMP-12 as a novel asthma biomarker associated with both inflammatory and remodeling phases of asthma [50]. Likewise, Louten and his colleagues demonstrated that MMP-12 expression was elevated in bronchoalveolar lavage cells and lung tissue in chronic murine asthma as well as in nonhuman primate asthma models [51]. Noticeably, MMP-12 levels decreased following dexamethasone treatment, indicating that MMP-12 may also serve as a therapeutic-response biomarker [51]. Clinical investigations further established the association between MMP-12 and asthma severity. Chaudhuri and his colleagues analyzed induced sputum samples from asthmatic patients with varying disease severities and reported increased sputum MMP-12 concentrations in smokers with asthma compared with healthy nonsmokers [52]. Their findings suggested that cigarette smoking may augment MMP-12-mediated airway injury in asthma, potentially contributing to accelerated lung-function decline [52]. Hinks and his colleagues performed multidimensional endotyping of severe asthma patients and identified distinct inflammatory clusters characterized by elevated sputum MMP-1, MMP-3, MMP-8, and MMP-12 levels [53]. Overexpression of MMP-12 was particularly associated with severe asthma phenotypes [53]. In 2020, Vieira and his colleagues reported that MMP-12 was overexpressed in obese asthmatic mice, together with an imbalance of MMP-2, MMP-8 and MMP-9, and accompanied by excessive ECM degradation and increased collagen I/III deposition [54]. In 2024, Liao and his colleagues provided proteomic evidence emphasizing the critical role of MMP-12 in neutrophil-dominant and steroid-insensitive asthma [55].

In the present study, we aimed to clarify the contribution of *MMP-12* genetic polymorphisms to asthma susceptibility in a genetically and geographically representative Taiwanese population. To the best of our knowledge, this investigation represents the first comprehensive analysis evaluating the relationship between *MMP-12* variants and asthma risk in Taiwan. Although neither the genotypic nor allelic distributions of *MMP-12* rs2276109 and rs652438 demonstrated statistically significant associations with overall asthma susceptibility in the primary susceptibility analyses (Table 2 and Table 3), several noteworthy findings emerged from subsequent stratified investigations. Because of the small number of rare homozygous genotype carriers, the corresponding effect estimates by stratified analysis should be interpreted cautiously. First, age-stratified analyses revealed that the impact of *MMP-12* rs652438 polymorphisms on asthma susceptibility appeared to be more evident among younger individuals (Table 4). Specifically, carriers of the variant genotypes exhibited significantly elevated asthma risk in subjects aged 25–40 years. In contrast, no corresponding association was observed in individuals older than 40 years. Interestingly, we propose a speculative yet biologically plausible explanation for this phenomenon. It is possible that the rs652438 variant genotypes (CT + CC) confer increased disease severity and poorer clinical outcomes across both age groups. However, among older individuals, the detrimental biological effects associated with these variant genotypes may lead to some uncertain exploratory consequences, thereby resulting in an apparent reduction in the proportion of CT and CC carriers among asthma patients older than 40 years. Indeed, the frequencies of the CT and CC genotypes among asthma patients markedly declined from 27.8% and 5.3% in the younger subgroup to 12.3% and 0% in the older subgroup, respectively, creating a misleading impression that the rs652438 variant might exert a protective effect in older populations (Table 4). Nevertheless, this hypothesis remains preliminary and requires validation in larger-scale cohorts, together with further mechanistic investigations addressing the biological consequences of distinct *MMP-12* genotypes. Second, gender-stratified analyses demonstrated no significant interaction between rs652438 polymorphisms and sex in modulating asthma susceptibility (Table 5), suggesting that the biological influence of this variant is unlikely to be sex-dependent in the investigated population. Similarly, equivalent subgroup analyses were also conducted for rs2276109; however, no statistically significant associations were identified in any stratified models. These findings collectively suggest that rs2276109 may exert only a limited contribution, if any, to asthma susceptibility or disease heterogeneity in this cohort. Most importantly, one of the major highlights of the present study is the significant association identified between *MMP-12* rs652438 polymorphisms and asthma severity (Table 6). Our data demonstrated that carriers of the rs652438 variant C allele, particularly individuals harboring the CT or CC genotypes, were more likely to develop more severe forms of asthma. These findings suggest that rs652438 may be associated with asthma severity. However, these observations should be considered exploratory and require validation in larger multicenter prospective studies before any clinical application. From a translational perspective, determination of *MMP-12* rs652438 genotypes may provide clinicians with valuable predictive information regarding disease severity at an early stage.

As early as 2010, Mukhopadhyay and his colleagues genotyped 1017 asthmatic and 1451 non-asthmatic subjects in the United Kingdom to investigate the association between *MMP-12* genotypes and asthma susceptibility [29]. They found that *MMP-12* rs652438 was significantly associated with asthma severity in children and young adults. Carriers of the *MMP-12* rs652438 CC genotype had a two-fold higher risk of greater asthma severity, and the same genotype was associated with significantly elevated asthma exacerbation risk [29]. Three years later, Yamaide and his colleagues further supported the involvement of *MMP-12* genotype in determining asthma susceptibility. They conducted a case–control study among 1047 asthmatic (containing 630 childhood and 417 adult) and 968 (containing 336 pediatric and 632 adult) non-asthmatic subjects, finding significant associations between the *MMP-12* rs652438 C allele and childhood asthma and combined childhood/adult asthma risk [39]. Notably, all participants were Japanese and therefore belonged to the East Asian population. This team has also provided evidence that MMP-12 siRNA treatment can significantly reduce IP-10 secretion from primary cultured normal human small airway epithelial cells following stimulation with IFN-β [39]. In 2018, Tacheva and his colleagues had just started to focus on examining the association of another *MMP-12* polymorphic site, rs2276109, with asthma risk prediction among 58 Bulgarian adults with bronchial asthma and 119 controls [40]. They found that the C allele and C-containing genotypes were less frequent in asthma patients, and carriers of CT/CC genotypes showed a significantly reduced risk of bronchial asthma [40]. Although their sample size is relatively small, their contribution is precious and remarkable.

In the current study, the genotypic distribution of *MMP-12* rs2276109 polymorphism in the control group conformed to HWE (*p* = 0.6819, Table 2), supporting the validity of our sample selection and genotyping methodology, similar to a comparative review of large-scale population data from the National Center for Biotechnology Information SNP database [56]. The 1000Genomes_30X official website reveals that the minor C allele occurs at an overall frequency of 5.3% among 6404 individuals globally, with substantial variation across racial/ethnic groups, including 3.2% among 1170 East Asians, 6.0% among 980 Americans, and 11.7% among 1266 individuals of European descent. In our study, the minor C allele frequency was 3.9% among the controls (Table 3), similar to the level observed among East Asian populations. In contrast, the C allele frequency in our population is markedly lower than that observed in populations of European origin. As for *MMP-12* rs652438, the allelic distribution of this variant in our control group showed a minor C allele frequency of 11.1% (Table 3). The official website reveals that the minor C allele occurs at an overall frequency of 7.2% among 448,558 individuals globally, with substantial variation across racial/ethnic groups, including 10.0% among 7026 East Asians, 18.0% among 50,490 African, 17.8% among 48,726 African Americans, and 5.4% among 334,738 individuals of European descent [57]. Let us look deeper into the perspective from the limited studies revealing the contribution of genotypes of *MMP-12* rs2276109 or rs652438 SNPs and asthma susceptibility from the United Kingdom, Bulgaria, and Japan [29,39,40]. The predictive marker for asthma susceptibility of *MMP-12* rs652438 C allele was found by a Japanese team in childhood asthma and combined childhood/adult asthma [39]. In the circumstance that adult asthma was analyzed alone, the association was not significant, consistent with ours (only adults aged larger than 25) of the same East Asian origin. The tentative conclusion is that rs652438 should be further validated for its becoming a qualified asthma predictor in adults. Interestingly, Mukhopadhyay’s team has similar findings to ours [29], supporting the feasibility of *MMP-12* rs652438 C allele for serving as an asthma severity predictor and clinical medication indicator not only in European but in East Asian populations.

From the asthma therapeutic viewpoint, selective inhibition of MMP-12 has shown promising prospects. Li and his colleagues identified a potent orally active MMP-12 inhibitor that significantly suppressed allergen-induced late-phase asthmatic responses in naturally sensitized sheep asthma models [58]. The study suggested that pharmacological targeting of MMP-12 may attenuate airway inflammation and remodeling while offering a disease-modifying strategy for asthma medication [58]. In a multicenter study, Magnussen and his colleagues have evaluated the safety and efficacy of an oral mixed MMP-9/MMP-12 inhibitor (AZD1236) in COPD patients [59]. Similarly, Dahl and his colleagues repeated the trial in next year [60], but the results seemed ineffective due to the short duration of treatment (6 weeks) in patients with initially stable disease and the fact that the drug effects were evaluated on top of concurrent controller medications [59,60]. Up to now, almost no published literature has ever existed on MMP-12 inhibitors in patients with asthma except one. As the website clinicaltrials.gov can be consulted, only one phase IIa proof-of-concept study with an MMP-12 inhibitor intervention in asthma (ClinicalTrials.gov ID: NCT03858686) has been recorded. The selective MMP-12 inhibitor Aderamastat (FP-025) demonstrated reductions in airway hyperresponsiveness, inflammatory-cell infiltration, fibrosis, and mucus production in murine allergic asthma models [61]. Our work stepped further to precise and personalized medication of asthma, showing that severe asthma patients may benefit from taking an MMP-12 inhibitor, such as FP-025, based on their *MMP-12* personal genotyping.

Mechanistically, MMP-12 appears to regulate several pathological features of asthma. MMP-12 promotes degradation of elastin and other ECM proteins, facilitating inflammatory-cell migration and airway remodeling [62]. MMP-12 also contributes to eosinophil and macrophage accumulation in allergen-challenged lungs and amplifies IL-13-mediated pathological responses [63]. Experimental studies demonstrated that MMP-12-deficient mice exhibit reduced airway eosinophilia, attenuated airway hyperresponsiveness, and diminished peribronchial fibrosis after allergen exposure [64]. These findings strongly support a causative role of MMP-12 in asthma pathophysiology. In 2023, Camargo and his colleagues found that IL-17 inhibition reduced airway hyperresponsiveness, inflammatory cells, cytokines, oxidative stress, and remodeling markers in an asthma–COPD overlap model. Importantly, anti-IL-17 treatment reduced MMP-12 together with MMP-9, TIMP-1, TGF-β, and collagen I, suggesting that MMP-12 may act downstream of IL-17-driven inflammatory remodeling [65]. More functional investigations are necessary to clarify the biological mechanisms underlying MMP-12 involvement in asthma etiology.

Despite these valuable findings, several limitations should be acknowledged. First, we did not assess MMP-12 expression levels in peripheral blood samples from either asthmatic patients or non-asthmatic healthy controls. Second, the study was performed within a single medical center, which may restrict the broader clinical applicability of our findings to other populations or ethnic groups. Third, several clinically relevant variables, such as body mass index, blood eosinophil counts, serum IgE levels, atopy status, and other comorbidities, were not systematically available for all participants. Fourth, relatively small numbers of participants carrying the rare homozygous genotypes were observed, particularly for variant genotypes in rs2276109 and rs652438. Consequently, the corresponding effect estimates may be unstable, especially in subgroup analyses. In addition, because multiple subgroup analyses were performed, the stratified findings should be considered exploratory and interpreted with caution. Independent validation in larger cohorts is required before firm conclusions can be drawn. Fifth, genotype–phenotype correlation and the detailed mechanisms by which MMP-12 mRNA/protein expression and/or enzymatic activity affect asthma severity require much more investigation. Lastly, although smoking status was included in the present analyses, information regarding occupational exposure, respiratory comorbidities other than asthma, and atopy/allergic sensitization was not systematically collected for all participants. Therefore, the potential confounding effects of these variables could not be evaluated and should be addressed in future studies. These aspects are also critical points for the precise and personalized application of MMP-12 inhibitors.

## 5. Conclusions

In conclusion, this study provides the first comprehensive evaluation of the association between *MMP-12* polymorphisms and asthma susceptibility and clinical phenotypes in a Taiwanese population. Overall, neither the genotypic nor allelic distributions of rs2276109 and rs652438 were significantly associated with asthma risk, indicating that these variants may not play a major role in determining overall asthma susceptibility. However, stratified analyses revealed important context-dependent effects. First, the rs652438 variant genotypes (CT and CC) were significantly associated with increased asthma risk among younger individuals aged 25 to 40 years, suggesting a potential age-specific genetic influence. Second, gender did not significantly modify the relationship between rs652438 and asthma susceptibility. Most noticeably, there is a significant association between rs652438 polymorphisms and asthma severity (Table 6). That is to say, carriers of the variant C allele at *MMP-12* rs652438 are prone to have a higher risk of developing more severe asthma. This finding suggests that rs652438 may serve as a clinically relevant genetic marker associated with asthma severity rather than a primary susceptibility locus. We should keep in mind that these findings require validation in larger cohorts with adequate numbers of rare genotype carriers. These findings suggest that rs652438 may represent a candidate genetic marker associated with asthma severity. Further validation in larger prospective studies and functional investigations is required before clinical application. Further functional studies are warranted to validate these observations and to elucidate the underlying biological mechanisms, ultimately supporting the development of precision medicine approaches targeting MMP-12 in the therapy and care for asthmatic patients.

## Figures and Tables

**Table 1:** Distributions of baseline characteristics among the 198 asthmatic patients and 453 non-asthmatic controls.

Character		Controls (N = 453)		Cases (N = 198)		*p*-Value ^a^
		N	%	N	%	
Age (years)	25–40	285	62.9%	133	67.2%	
	>40	168	37.1%	65	32.8%	0.2972
Gender	Male	190	41.9%	83	41.9%	
	Female	263	58.1%	115	58.1%	0.9956
Smoking status	Never	326	72.0%	139	70.2%	
	Ever	127	28.0%	59	29.8%	0.7161
Pulmonary functions	(mean ± SD)					
	FEV1/FVC (%)	80.8 ± 8.1		62.0 ± 13.0		<0.0001
	FEV1%	92.9 ± 5.8		69.1 ± 12.9		<0.0001
Symptoms severity						
	1 (mildest)			60	30.3%	
	2			65	32.8%	
	3			34	17.2%	
	4 (severest)			39	19.7%	

Abbreviation: FEV1, forced expiratory volume in the first second; FVC, forced vital capacity; FEV1%, percent of predicted FEV1. ^a^ Categorical variables were analyzed using the chi-square test, whereas continuous variables (FEV1/FVC and FEV1%) were analyzed using the unpaired *Student’s t*-test. The four treatment groups reflect treatment intensity according to the contemporaneous GINA recommendations used during patient recruitment (2008–2010). The definition of the four severity groups, based on the level of treatment required according to the GINA recommendations as detailed in Section 2.1.

**Table 2:** *MMP-12* genotypes among the 198 asthmatic patients and 453 non-asthmatic controls.

Genotype	Cases		Controls		OR (95% CI) ^a^	OR (95% CI) ^a^	*p*-Value ^b^
	N	%	N	%			
rs2276109							
TT	180	90.9%	419	92.5%	1.00 (Reference)	1.00 (Reference)	
CT	15	7.6%	33	7.3%	1.06 (0.56–2.00)	1.09 (0.61–1.93)	0.9913
CC	3	1.5%	1	0.2%	6.98 (0.72–67.59)	6.13 (0.74–56.41)	0.0857
*p*_trend_							0.2622
*p*_HWE_							0.6819
Carrier comparison							
TT + CT	195	98.5%	452	99.8%	1.00 (Reference)	1.00 (Reference)	
CC	3	1.5%	1	0.2%	6.95 (0.72–67.27)	6.04 (0.75–58.86)	0.0862
TT	180	90.9%	419	92.5%	1.00 (Reference)	1.00 (Reference)	
CT + CC	18	9.1%	34	7.5%	1.23 (0.68–2.24)	1.19 (0.71–2.03)	0.5966
rs652438							
TT	146	73.8%	360	79.4%	1.00 (Reference)	1.00 (Reference)	
CT	45	22.7%	85	18.8%	1.31 (0.87–1.97)	1.26 (0.90–1.89)	0.2416
CC	7	3.5%	8	1.8%	2.16 (0.77–6.06)	1.98 (0.79–5.53)	0.2281
*p*_trend_							0.0672
*p*_HWE_							0.2609
Carrier comparison							
TT + CT	191	96.5%	445	98.2%	1.00 (Reference)	1.00 (Reference)	
CC	7	3.5%	8	1.8%	2.04 (0.73–5.70)	1.95 (0.68–4.93)	0.2712
TT	146	73.8%	360	79.4%	1.00 (Reference)	1.00 (Reference)	
CT + CC	52	26.2%	93	20.6%	1.38 (0.93–2.04)	1.31 (0.91–1.88)	0.1298

N, number; ^a^ adjusted for age, gender, and smoking status; ^b^ based on chi-square test with Yates’ correction (n ≥ 5) or Fisher’s exact test (n < 5); OR, odds ratio; CI, confidence interval; *p*_trend:_ *p*-value for Cochran–Armitage trend analysis; *p*_HWE_, *p*-value for Hardy–Weinberg equilibrium analysis.

**Table 3:** Distribution of allelic frequencies for *MMP-12* rs2276109 and rs652438 among the 198 asthmatic patients and 453 non-asthmatic controls.

Allele	Cases, N	%	Controls, N	%	OR (95% CI) ^a^	OR (95% CI) ^a^	*p*-Value ^b^
rs2276109							
T	375	94.7%	871	96.1%	1.00 (Reference)	1.00 (Reference)	
C	21	5.3%	35	3.9%	1.39 (0.80–2.43)	1.33 (0.81–2.14)	0.3032
rs652438							
T	337	85.1%	805	88.9%	1.00 (Reference)	1.00 (Reference)	
C	59	14.9%	101	11.1%	1.40 (0.99–1.97)	1.33 (0.95–1.87)	0.0711

N: number; ^a^ adjusted for age, gender, and smoking status; ^b^ based on chi-square test with Yates’ correction; OR, odds ratio; CI, confidence interval.

**Table 4:** *MMP-12* rs652438 genotypes in asthma risk after stratification by age.

Genotype	Younger (25–40), N		OR (95% CI) ^a^	*p*-Value ^b^	Elder (>40), N		OR (95% CI) ^a^	*p*-Value ^b^
	Controls	Cases			Controls	Cases		
TT	229	89	1.00 (Ref)		129	57	1.00 (Ref)	
CT	51	37	**1.86** **(1.14–3.04)**	0.0100 *	36	8	0.50 (0.22–1.15)	0.1429
CC	5	7	**3.60 (1.11–11.65)**	0.0494 *	3	0	0.32 (0.02–6.33)	0.5550
Total	285	133			168	65		
*p*_trend_				0.0014 *				0.0487 *

N: number; Ref: reference; ^a^ Haldane–Anscombe correction was used for OR and 95% CI calculation when the number of specific cell is zero; ^b^ based on chi-square test with Yates’ correction (n ≥ 5) or Fisher’s exact test (n < 5); CI, confidence interval; OR, odds ratio; *p*_trend,_ *p*-value for trend analysis by Cochran–Armitage trend test; * statistical significance is set as *p*-value less than 0.05.

**Table 5:** *MMP-12* rs652438 genotypes in asthma risk after stratification by gender.

Genotype	Males, N		OR (95% CI)	*p*-Value ^a^	Females, N		OR (95% CI)	*p*-Value ^a^
	Controls	Cases			Controls	Cases		
TT	152	60	1.00 (Ref)		208	86	1.00 (Ref)	
CT	34	19	1.42 (0.75–2.67)	0.3647	51	26	1.23 (0.72–2.11)	0.5295
CC	4	4	2.53 (0.61–10.46)	0.2346	4	3	1.81 (0.40–8.28)	0.4257
Total	190	83			263	115		
*p*_trend_				0.1112				0.3036

N: number; Ref: reference; ^a^ based on chi-square test with Fisher’s exact test; CI, confidence interval; OR, odds ratio; *p*_trend,_ *p*-value for trend analysis by Cochran–Armitage trend test.

**Table 6:** Genotype frequencies of *MMP-12* rs652438 genotypes in patients with milder and more severe asthma.

Genotype	Milder Asthma (N) ^a^	More Severe Asthma (N) ^b^	OR (95%CI) ^a^	OR (95% CI) ^c^	*p*-Value ^d^
TT	106 (79.7%)	40 (61.5%)	1.00 (Reference)	1.00 (Reference)	
CT	24 (18.1%)	21 (32.3%)	**2.32 (1.16–4.62)**	2.27 (1.18–4.23)	0.0250 *
CC	3 (2.2%)	4 (6.2%)	3.53 (0.75–16.49)	3.34 (0.81–12.14)	0.1054
*p*_trend_					0.0059 *
TT + CT	130 (97.8%)	61 (93.8%)	1.00 (Reference)	1.00 (Reference)	
CC	3 (2.2%)	4 (6.2%)	2.84 (0.62–13.09)	2.53 (0.70–11.84)	0.2201
TT	106 (79.7%)	40 (61.5%)	1.00 (Reference)	1.00 (Reference)	
CT + CC	27 (20.3%)	25 (38.5%)	**2.45 (1.28–4.72)**	2.34 (1.23–4.17)	0.0106 *

N: number; CI: Confidence interval; OR: odds ratio. ^a^ milder asthma: severe stages 1 and 2; ^b^ more severe asthma: severe stages 3 and 4; ^c^ adjusted for age, gender, and smoking status; ^d^ based on chi-square test with Yates’ correction (n ≥ 5) or Fisher’s exact test (n < 5); *p*_trend,_ *p*-value for trend analysis by Cochran-Armitage trend test; * statistical significance is set as *p*-value less than 0.05.

## Data Availability

The data that support the findings of this study are available on request from the corresponding author. The data are not publicly available due to privacy or ethical restrictions.

## Abbreviations

The following abbreviations are used in this manuscript:

VEGF	Vascular endothelial growth factor
IL-4	Interleukin-4
IL-12	Interleukin-12
IL-13	Interleukin-13
ADAM-33	A disintegrin and metalloproteinase-33
PM2.5	Particulate matter
MMPs	Matrix metalloproteinases
ECM	Extracellular matrix
SNPs	Single nucleotide polymorphisms
AP-1	Activator protein-1
COPD	Chronic obstructive pulmonary disease
GINA	Global initiative for asthma
PCR-RFLP	Polymerase chain reaction–restriction fragment length polymorphism
HWE	Hardy–Weinberg equilibrium
ORs	Odds ratios
CIs	Confidence intervals
AZD1236	MMP-9/MMP-12 inhibitor
FP-025	MMP-12 inhibitor
